# 2-(4-Bromo­phen­yl)-*N*-(2-methoxy­phen­yl)acetamide

**DOI:** 10.1107/S1600536809051812

**Published:** 2009-12-09

**Authors:** Zhu-Ping Xiao, Yu-Zhu Ouyang, Shi-Dong Qin, Tian Xie, Jia Yang

**Affiliations:** a College of Chemistry & Chemical Engineering, Jishou University, Jishou 416000, People’s Republic of China

## Abstract

In the title compound, C_15_H_14_BrNO_2_, the 4-bromo­phenyl fragment makes a dihedral angle of 76.55 (17)° with the acetamide unit and the dihedral angle between the two benzene rings is 50.88 (14)°. In the crystal structure, inter­molecular N—H⋯O hydrogen bonds and C—H⋯π contacts connect the mol­ecules, forming chains propagating in [100].

## Related literature

For background to phenyl­acetamide derivatives as potential anti­microbial agents, see: Mijin & Marinković (2006 ▶); Mijin *et al.* (2008 ▶).
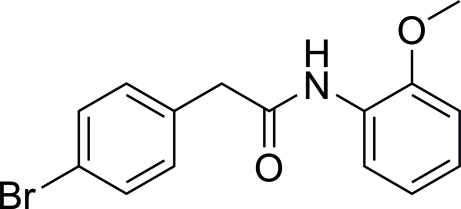


## Experimental

### 

#### Crystal data

C_15_H_14_BrNO_2_
*M* *_r_* = 320.18
Triclinic, 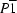

*a* = 4.851 (4) Å
*b* = 12.083 (10) Å
*c* = 12.265 (10) Å
α = 74.61 (3)°
β = 87.47 (3)°
γ = 85.18 (3)°
*V* = 690.5 (10) Å^3^
*Z* = 2
Mo *K*α radiation
μ = 2.97 mm^−1^
*T* = 296 K
0.25 × 0.20 × 0.10 mm

#### Data collection

Bruker SMART APEX CCD diffractometer
Absorption correction: multi-scan (*SADABS*; Sheldrick, 1996 ▶) *T*_min_ = 0.524, *T*_max_ = 0.755
3678 measured reflections
2642 independent reflections
1441 reflections with *I* > 2σ(*I*)
*R*_int_ = 0.027

#### Refinement

*R*[*F* ^2^ > 2σ(*F* ^2^)] = 0.053
*wR*(*F* ^2^) = 0.133
*S* = 1.01
2642 reflections
177 parameters
H atoms treated by a mixture of independent and constrained refinement
Δρ_max_ = 0.79 e Å^−3^
Δρ_min_ = −0.62 e Å^−3^

### 

Data collection: *SMART* (Bruker, 2007 ▶); cell refinement: *SAINT* (Bruker, 2007 ▶); data reduction: *SAINT*; program(s) used to solve structure: *SHELXS97* (Sheldrick, 2008 ▶); program(s) used to refine structure: *SHELXL97* (Sheldrick, 2008 ▶); molecular graphics: *SHELXTL* (Sheldrick, 2008 ▶); software used to prepare material for publication: *SHELXL97*.

## Supplementary Material

Crystal structure: contains datablocks global, I. DOI: 10.1107/S1600536809051812/hb5263sup1.cif

Structure factors: contains datablocks I. DOI: 10.1107/S1600536809051812/hb5263Isup2.hkl

Additional supplementary materials:  crystallographic information; 3D view; checkCIF report

## Figures and Tables

**Table 1:** Hydrogen-bond geometry (Å, °)

*D*—H⋯*A*	*D*—H	H⋯*A*	*D*⋯*A*	*D*—H⋯*A*
N1—H1⋯O2^i^	0.81 (5)	2.13 (5)	2.912 (6)	160 (5)
C15—H15*C*⋯*Cg*1^i^	0.96	2.86	3.617 (7)	137

Symmetry code: (i) 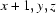. *Cg*1 is the centroid of the C9–C14 ring.

**Fig. 1.**
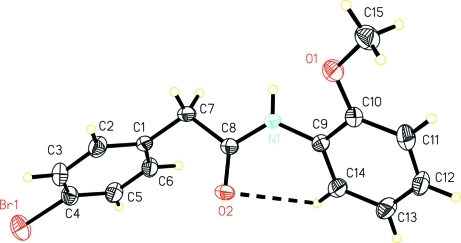
Molecular structure of (I) with displacement ellipsoids drawn at the 30% probability level.

**Fig. 2.**
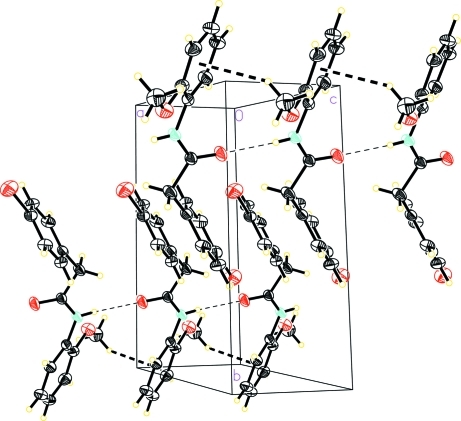
Part of the crystal structure of (I) with hydrogen bonds indicated by thin dashed lines and C—H···π contacts shown as thick dashed lines.

## supplementary crystallographic information

### Comment

N-Substituted 2-phenylacetamides are very interesting compounds because of their structural similarity to the lateral chain of natural benzylpenicillin (Mijin *et al.*, 2008; Mijin *et al.*, 2006). As part of our work involving the synthesis of a series of phenylacetamide derivatives for antimicrobial activity screening, we report herein the crystal structure of the title phenylacetamide derivative (I).

The molecular structure of the title compound is shown in Fig. 1. The two benzene rings in form a dihedral angle of 50.88 (14) °. The acetamide fragment makes a dihedral angle of 76.55 (17) ° with the *p*-bromophenyl group [Br1/C1-C6]. The two benzene rings and the acetamide skeleton show a W-shape figuration. In the crystal structure, intermolecular N—H···O  together with C—H···π contacts connect molecules to form an infinite line running along the crystallographic a-axis direction (see Fig. 2).

### Experimental

1.17 g (5 mmol) of 4-bromophenylacetyl chloride and 0.62 g (5 mmol) of 2-methoxyaniline were dissolved into 20 mL of fresh distilled CH_2_Cl_2_. The mixture was stirred in the present of triethylamine at 273 K for about 3 h. The contents were poured into 100 ml of ice-cold aqueous hydrochloride acid (w % = 5%) with stirring, which was extracted thrice with EtOAc. The EtOAc solution was washed with aqueous saturated NaHCO_3_ and brine, dried and concentrated under reduced pressure to give the product as a light yellow solid which on crystallization from EtOAc-petrolium ether gave colourless blocks of (I).

### Refinement

The H atom bonded to N1 was located in a difference Fourier map. All other H atoms were placed in geometrically idealized positions and constrained to ride on their parent atoms with C—H of 0.93 Å for the aromatic atoms, 0.97 Å for the CH_2_ groups and 0.96 Å for the CH_3_ groups. *U*_iso_(H) values were set at 1.2 times *U*_eq_(C) for aromatic C and CH_2_ groups, and 1.5 times for CH_3_ groups.

### Crystal data

**Table:**

C_15_H_14_BrNO_2_	*Z* = 2
*M**_r_* = 320.18	*F*(000) = 324
Triclinic, *P*1	*D*_x_ = 1.540 Mg m^−^^3^
Hall symbol: -P 1	Mo *K*α radiation, λ = 0.71073 Å
*a* = 4.851 (4) Å	Cell parameters from 1389 reflections
*b* = 12.083 (10) Å	θ = 2.4–26.0°
*c* = 12.265 (10) Å	µ = 2.97 mm^−^^1^
α = 74.61 (3)°	*T* = 296 K
β = 87.47 (3)°	Block, colourless
γ = 85.18 (3)°	0.25 × 0.20 × 0.10 mm
*V* = 690.5 (10) Å^3^	

### Data collection

**Table:**

Bruker SMART APEX CCD diffractometer	2642 independent reflections
Radiation source: fine-focus sealed tube	1441 reflections with *I* > 2σ(*I*)
graphite	*R*_int_ = 0.027
φ and ω scan	θ_max_ = 26.0°, θ_min_ = 1.7°
Absorption correction: multi-scan (*SADABS*; Sheldrick, 1996)	*h* = −5→5
*T*_min_ = 0.524, *T*_max_ = 0.755	*k* = −10→14
3678 measured reflections	*l* = −14→15

### Refinement

**Table:**

Refinement on *F*^2^	Primary atom site location: structure-invariant direct methods
Least-squares matrix: full	Secondary atom site location: difference Fourier map
*R*[*F*^2^ > 2σ(*F*^2^)] = 0.053	Hydrogen site location: inferred from neighbouring sites
*wR*(*F*^2^) = 0.133	H atoms treated by a mixture of independent and constrained refinement
*S* = 1.01	*w* = 1/[σ^2^(*F*_o_^2^) + (0.0528*P*)^2^ + 0.5023*P*] where *P* = (*F*_o_^2^ + 2*F*_c_^2^)/3
2642 reflections	(Δ/σ)_max_ < 0.001
177 parameters	Δρ_max_ = 0.79 e Å^−^^3^
0 restraints	Δρ_min_ = −0.61 e Å^−^^3^

### Special details

**Table:**

Geometry. All esds (except the esd in the dihedral angle between two l.s. planes) are estimated using the full covariance matrix. The cell esds are taken into account individually in the estimation of esds in distances, angles and torsion angles; correlations between esds in cell parameters are only used when they are defined by crystal symmetry. An approximate (isotropic) treatment of cell esds is used for estimating esds involving l.s. planes.
Refinement. Refinement of F^2^ against ALL reflections. The weighted R-factor wR and goodness of fit S are based on F^2^, conventional R-factors R are based on F, with F set to zero for negative F^2^. The threshold expression of F^2^ > 2sigma(F^2^) is used only for calculating R-factors(gt) etc. and is not relevant to the choice of reflections for refinement. R-factors based on F^2^ are statistically about twice as large as those based on F, and R- factors based on ALL data will be even larger.

### Fractional atomic coordinates and isotropic or equivalent isotropic displacement parameters (Å^2^)

**Table:**

	*x*	*y*	*z*	*U*_iso_*/*U*_eq_	
Br1	−0.42219 (14)	0.65388 (5)	0.56611 (5)	0.0755 (3)	
C1	0.2053 (9)	0.4363 (4)	0.8358 (4)	0.0410 (12)	
C2	0.0778 (11)	0.5420 (4)	0.8452 (4)	0.0501 (14)	
H2	0.1226	0.5707	0.9049	0.060*	
C3	−0.1146 (11)	0.6050 (4)	0.7670 (4)	0.0519 (14)	
H3	−0.2009	0.6741	0.7753	0.062*	
C4	−0.1743 (10)	0.5640 (4)	0.6782 (4)	0.0469 (13)	
C5	−0.0535 (11)	0.4593 (4)	0.6656 (4)	0.0515 (14)	
H5	−0.0979	0.4315	0.6052	0.062*	
C6	0.1339 (10)	0.3977 (4)	0.7450 (4)	0.0493 (14)	
H6	0.2152	0.3277	0.7371	0.059*	
C7	0.4103 (10)	0.3672 (4)	0.9209 (4)	0.0525 (14)	
H7A	0.4654	0.4153	0.9669	0.063*	
H7B	0.5741	0.3452	0.8811	0.063*	
C8	0.2970 (10)	0.2594 (4)	0.9980 (4)	0.0394 (12)	
C9	0.4355 (9)	0.0797 (4)	1.1414 (4)	0.0393 (12)	
C10	0.5674 (10)	0.0471 (4)	1.2445 (4)	0.0429 (12)	
C11	0.5351 (11)	−0.0618 (4)	1.3177 (4)	0.0543 (15)	
H11	0.6274	−0.0846	1.3858	0.065*	
C12	0.3679 (12)	−0.1345 (4)	1.2889 (5)	0.0589 (15)	
H12	0.3461	−0.2064	1.3383	0.071*	
C13	0.2316 (12)	−0.1032 (4)	1.1884 (5)	0.0581 (15)	
H13	0.1152	−0.1530	1.1708	0.070*	
C14	0.2681 (10)	0.0036 (4)	1.1129 (4)	0.0465 (13)	
H14	0.1811	0.0240	1.0436	0.056*	
C15	0.8674 (12)	0.0996 (5)	1.3693 (5)	0.0636 (16)	
H15A	0.7386	0.0774	1.4315	0.095*	
H15B	0.9569	0.1654	1.3758	0.095*	
H15C	1.0038	0.0369	1.3706	0.095*	
H1	0.645 (11)	0.205 (4)	1.063 (4)	0.055 (17)*	
N1	0.4851 (9)	0.1880 (3)	1.0651 (3)	0.0432 (11)	
O1	0.7238 (7)	0.1276 (3)	1.2659 (3)	0.0558 (10)	
O2	0.0524 (7)	0.2422 (3)	0.9995 (3)	0.0567 (10)	

### Atomic displacement parameters (Å^2^)

**Table:**

	*U*^11^	*U*^22^	*U*^33^	*U*^12^	*U*^13^	*U*^23^
Br1	0.0798 (5)	0.0708 (4)	0.0623 (4)	0.0148 (3)	−0.0253 (3)	0.0037 (3)
C1	0.033 (3)	0.035 (3)	0.050 (3)	−0.009 (2)	−0.005 (2)	0.000 (2)
C2	0.061 (4)	0.039 (3)	0.046 (3)	−0.009 (3)	−0.006 (3)	−0.003 (2)
C3	0.064 (4)	0.035 (3)	0.053 (3)	0.006 (3)	−0.004 (3)	−0.008 (2)
C4	0.048 (3)	0.043 (3)	0.041 (3)	−0.001 (2)	−0.003 (2)	0.004 (2)
C5	0.062 (4)	0.043 (3)	0.050 (3)	−0.005 (3)	−0.007 (3)	−0.011 (2)
C6	0.049 (3)	0.035 (3)	0.059 (4)	0.000 (2)	0.003 (3)	−0.007 (2)
C7	0.036 (3)	0.047 (3)	0.065 (4)	−0.014 (2)	−0.015 (3)	0.008 (3)
C8	0.033 (3)	0.040 (3)	0.042 (3)	−0.006 (2)	−0.004 (2)	−0.005 (2)
C9	0.034 (3)	0.037 (3)	0.043 (3)	−0.005 (2)	0.002 (2)	−0.004 (2)
C10	0.042 (3)	0.035 (3)	0.049 (3)	0.000 (2)	−0.004 (2)	−0.008 (2)
C11	0.062 (4)	0.049 (3)	0.044 (3)	0.008 (3)	−0.004 (3)	−0.001 (3)
C12	0.063 (4)	0.033 (3)	0.069 (4)	−0.002 (3)	0.003 (3)	0.004 (3)
C13	0.061 (4)	0.037 (3)	0.078 (4)	−0.012 (3)	0.000 (3)	−0.015 (3)
C14	0.047 (3)	0.043 (3)	0.048 (3)	−0.009 (2)	−0.005 (3)	−0.008 (2)
C15	0.065 (4)	0.068 (4)	0.058 (4)	−0.001 (3)	−0.018 (3)	−0.015 (3)
N1	0.031 (3)	0.038 (2)	0.053 (3)	−0.011 (2)	−0.010 (2)	0.0044 (19)
O1	0.061 (2)	0.054 (2)	0.049 (2)	−0.0071 (18)	−0.0191 (18)	−0.0043 (17)
O2	0.029 (2)	0.056 (2)	0.072 (3)	−0.0111 (17)	−0.0083 (17)	0.0096 (18)

### Geometric parameters (Å, °)

**Table:**

Br1—C4	1.910 (5)	C9—C10	1.388 (7)
C1—C6	1.383 (7)	C9—C14	1.397 (7)
C1—C2	1.403 (7)	C9—N1	1.424 (6)
C1—C7	1.505 (6)	C10—O1	1.367 (6)
C2—C3	1.394 (7)	C10—C11	1.399 (7)
C2—H2	0.9300	C11—C12	1.365 (8)
C3—C4	1.361 (7)	C11—H11	0.9300
C3—H3	0.9300	C12—C13	1.372 (7)
C4—C5	1.393 (7)	C12—H12	0.9300
C5—C6	1.381 (7)	C13—C14	1.394 (7)
C5—H5	0.9300	C13—H13	0.9300
C6—H6	0.9300	C14—H14	0.9300
C7—C8	1.521 (6)	C15—O1	1.421 (6)
C7—H7A	0.9700	C15—H15A	0.9600
C7—H7B	0.9700	C15—H15B	0.9600
C8—O2	1.220 (5)	C15—H15C	0.9600
C8—N1	1.346 (6)	N1—H1	0.81 (5)
			
C6—C1—C2	117.2 (4)	C10—C9—N1	119.0 (4)
C6—C1—C7	121.3 (5)	C14—C9—N1	121.6 (4)
C2—C1—C7	121.6 (5)	O1—C10—C9	115.3 (4)
C3—C2—C1	121.4 (5)	O1—C10—C11	124.8 (5)
C3—C2—H2	119.3	C9—C10—C11	119.9 (5)
C1—C2—H2	119.3	C12—C11—C10	119.9 (5)
C4—C3—C2	119.0 (5)	C12—C11—H11	120.1
C4—C3—H3	120.5	C10—C11—H11	120.1
C2—C3—H3	120.5	C11—C12—C13	121.1 (5)
C3—C4—C5	121.6 (5)	C11—C12—H12	119.4
C3—C4—Br1	119.3 (4)	C13—C12—H12	119.4
C5—C4—Br1	119.1 (4)	C12—C13—C14	119.8 (5)
C6—C5—C4	118.3 (5)	C12—C13—H13	120.1
C6—C5—H5	120.9	C14—C13—H13	120.1
C4—C5—H5	120.9	C13—C14—C9	119.9 (5)
C5—C6—C1	122.5 (5)	C13—C14—H14	120.0
C5—C6—H6	118.7	C9—C14—H14	120.0
C1—C6—H6	118.7	O1—C15—H15A	109.5
C1—C7—C8	113.2 (4)	O1—C15—H15B	109.5
C1—C7—H7A	108.9	H15A—C15—H15B	109.5
C8—C7—H7A	108.9	O1—C15—H15C	109.5
C1—C7—H7B	108.9	H15A—C15—H15C	109.5
C8—C7—H7B	108.9	H15B—C15—H15C	109.5
H7A—C7—H7B	107.7	C8—N1—C9	126.0 (4)
O2—C8—N1	123.6 (4)	C8—N1—H1	120 (4)
O2—C8—C7	121.6 (4)	C9—N1—H1	114 (4)
N1—C8—C7	114.8 (4)	C10—O1—C15	118.3 (4)
C10—C9—C14	119.3 (4)		
			
C6—C1—C2—C3	0.6 (7)	C14—C9—C10—C11	0.9 (7)
C7—C1—C2—C3	−179.2 (4)	N1—C9—C10—C11	−176.1 (4)
C1—C2—C3—C4	−1.6 (7)	O1—C10—C11—C12	178.4 (5)
C2—C3—C4—C5	1.8 (8)	C9—C10—C11—C12	−1.8 (8)
C2—C3—C4—Br1	−176.6 (4)	C10—C11—C12—C13	0.6 (8)
C3—C4—C5—C6	−1.0 (8)	C11—C12—C13—C14	1.4 (8)
Br1—C4—C5—C6	177.4 (4)	C12—C13—C14—C9	−2.2 (8)
C4—C5—C6—C1	−0.1 (7)	C10—C9—C14—C13	1.0 (7)
C2—C1—C6—C5	0.2 (7)	N1—C9—C14—C13	178.0 (5)
C7—C1—C6—C5	180.0 (4)	O2—C8—N1—C9	4.5 (8)
C6—C1—C7—C8	−71.8 (6)	C7—C8—N1—C9	−177.4 (5)
C2—C1—C7—C8	107.9 (5)	C10—C9—N1—C8	−142.9 (5)
C1—C7—C8—O2	−10.1 (7)	C14—C9—N1—C8	40.2 (7)
C1—C7—C8—N1	171.8 (4)	C9—C10—O1—C15	−179.3 (4)
C14—C9—C10—O1	−179.2 (4)	C11—C10—O1—C15	0.6 (7)
N1—C9—C10—O1	3.8 (7)		

### Hydrogen-bond geometry (Å, °)

**Table:**

*D*—H···*A*	*D*—H	H···*A*	*D*···*A*	*D*—H···*A*
N1—H1···O2^i^	0.81 (5)	2.13 (5)	2.912 (6)	160 (5)
C15—H15C···Cg1^i^	0.96	2.86	3.617 (7)	137

Symmetry codes: (i) *x*+1, *y*, *z*.
